# Initiating systemic capacity development for leadership from the bottom-up: a realist evaluation of a leadership innovation in a South African health district

**DOI:** 10.1093/heapol/czae099

**Published:** 2024-10-30

**Authors:** Marsha Orgill, Bruno Marchal, Bronwyn Harris, Lucy Gilson

**Affiliations:** Children’s Institute, Department of Paediatrics and Child Health, University of Cape Town, Rondebosch 7700, South Africa; Institute of Tropical Medicine, Antwerp 2000, Belgium; Centre for Health Policy, University of the Witwatersrand, Johannesburg 2017, South Africa; Division of Health Sciences, Warwick University, Coventry CV4 7AL, United Kingdom; Health Policy and Systems Division, Department of Public Health, University of Cape Town, 7700, South Africa; Department of Global Health and Development, London School of Hygiene and Tropical Medicine, London WC1E 7HT, United Kingdom

**Keywords:** Manager, capacity, capacity development, leadership, district health system, bottom-up implementation, systemic capacity

## Abstract

The need for leadership within district health systems is critical for the effective delivery of services and for inter-sectoral collaboration for health. Leadership capacity development (LCD) has not, however, been prioritized within health systems, and the systemic capacity (i.e. roles, structures and processes) that is needed to develop managers who can lead is not always in place. This paper aims to contribute to understanding how to build such capacity, considering a relevant bottom-up innovation. We observed, in the period 2013–15, the emergent implementation of this innovation (a ‘Leadership Commission’) in a South African health district. What started out as an effort to train individual leaders evolved into the development of systemic capacity for LCD. We adopted realist evaluation as the main methodological approach, as well as case study design, and we first developed a programme theory of the internally driven LCD initiative, through a round of interviews with senior managers. We then tested the programme theory drawing on 14 in-depth interviews and field notes of meetings and processes. Our analysis suggests that building systemic capacity for LCD requires leadership to be expressed as a strategic priority by those with positional authority and that bottom-up LCD requires institutional commitment through strengthening routine structures or creating new ones. The ability to leverage existing resources is another key element of systemic capacity. The mechanisms that enable bottom-up capacity development include tacit and experiential knowledge, sensemaking, systems thinking and trust between, and motivation of, those tasked with leading LCD. Leadership development is constrained by increased workloads for those involved as the prioritization of leadership becomes simply an additional task, and sustainability challenges are likely in the absence of additional resources for bottom-up innovation.

Key messages

- Leadership is a critical part of district health system functioning, and leadership capacity development can be constructed as an internal emergent process.
- Leadership must be recognized as a strategic priority alongside traditional disease or programmatic priorities.
- Systems leadership is required to address complex challenges in complex systems.
- Key mechanisms for supporting systemic capacity development from the bottom up include clarification of roles, structures and processes, tacit and experiential knowledge, sensegiving, sensemaking, shared beliefs and motivation, boundary spanners, champions, positional power, expressed commitment and institutional commitment.

## Introduction

The district health system (DHS) is a critical organizational level in any health system, as it is the platform for both the delivery of primary health care (PHC) and coordinating action with societal actors and across government departments (World Health Organisation, 1988; Van Lerberghe, 2008). Strengthening district leadership and management is widely recognized as vital to strengthening overall health system functioning and performance (Tanner, 2005; World Health Organization, 2007; Van Lerberghe, 2008; Doherty *et al*., 2018).

Health managers, including at the district level, not only have to grapple with the operational tasks of organizing, planning and budgeting, but they are also increasingly required to confront complex challenges in their daily work, such as epidemiological transitions and efforts to achieve universal coverage (Reich *et al*., 2016a; Belrhiti *et al*., 2018; Figueroa *et al*., 2019). Health systems are widely recognized to be complex adaptive systems. Navigating complex challenges in health systems requires being able to lead people at all levels of a system to take collective action towards shared goals (World Health Organization, 2016; Bigland *et al*., 2020). Such experiences have driven a shift in wider leadership discourse from concern for individual leaders’ characteristics and traits to thinking about leadership as distributed (Gronn, 2002), participatory (World Health Organization, 2016) and a relational and interactive practice (Cleary *et al*., 2018). These new leadership ideas emphasize team working and collective forms of leadership and see leadership as an organizational capability rather than an individual competence. In this sense, leadership capacity can be seen as an emergent property evolving within a system that can be facilitated through explicit or implicit workplace-based learning processes (Choonara *et al*., 2017; Martineau *et al*., 2018). There is, therefore, growing recognition that ‘strengthening health leadership is a system-wide reform that requires intervention at individual, team and system levels’ (World Health Organization, 2016; Reich *et al*., 2016b; Gilson and Agyepong, 2018) and that capacity development entails more than individual training. Yet, leadership capacity development (LCD) in health systems is mostly targeted at individuals (Johnson *et al*., 2021) and often entails a mix of on-the-job training and externally provided interventions (Curry *et al*., 2012; Daire *et al*., 2014; Chunharas and Davies, 2016; Belrhiti *et al*., 2018; Johnson *et al*., 2021), including in DHSs (Daire and Gilson, 2014; Cleary *et al*., 2018; Nzinga *et al*., 2021). Supporting individual training is typically viewed as an internal human resource development task, situated as a function of human resource departments, but LCD is rarely seen as an organizational priority. Little is known about internally driven LCD at the health district level.

A DHS was first initiated in South Africa in 1995, driven by the desire and goal to reduce the legacy of fragmentation and geographic inequity in access inherited from the previous apartheid government. The intention was to centre communities and a PHC approach, and the DHS was legally established in South Africa in 2003 (Gilson *et al*., 1996; Van Rensburg, 2012). Within a wider programme of district and PHC strengthening in 2012–17 (Pillay and Barron, 2012; Oboirien *et al*., 2014; Schneider *et al*., 2014), the South African National Department of Health introduced a series of innovations to strengthen the DHS in preparation for the introduction of proposed National Health Insurance reforms (SA NDoH, 2011; Matsoso and Fryatt, 2013).^1^ The South African National Department of Health selected 10 health district sites as ‘National Health Insurance pilot sites’, all of which were identified as most in need of improvement (Health-E News, 2012; Khumalo, 2012). The capacity of district management teams to implement the proposed reforms was seen as crucial to National Health Insurance piloting success as these managers provide oversight and direction on the PHC platform. While gains have been made in establishing the DHS in South Africa, management capacity has been noted as in need of improvement (Coovadia *et al*., 2009; Doherty *et al*., 2018). For both of these reasons, management strengthening development was identified as a key priority by the Minister of Health as part of the National Health Insurance piloting reforms (SA NDoH, 2011).

Box 1. **The bottom-up innovation for LCD**

In 2013, a new DM and her five top managers identified six strategic priorities for the district, called ‘commissions’, in the annual planning retreat. For the first time, leadership—this was the term actually used—was identified as a strategic priority in the district. The bottom-up innovation considered in this case is the ‘Leadership Commission’. This Commission was not a structure, but rather an expressed statement signalling the importance of LCD. The goal was to harness managers who could lead and transform the health system in the future. The meaning of leadership was not fully specified. Rather, ideas about how to develop leadership emerged through the process. The original premise of the Leadership Commission was to develop the capacity of individuals to lead, but over time it was recognized that the systemic capacity at the local level to implement LCD initiatives was limited: there were no formal structures and processes in place that specifically targeted and/or budgeted for LCD. Subsequently, efforts were made to set up structures and processes in the district to give ‘life’ to the Leadership Commission.

As part of this mandate, bottom-up strategies were developed by some district managers (DMs) (Orgill *et al*., 2021), and in the district of focus in this paper, the DM chose to focus specifically on ‘leadership capacity development’ (Box 1). In 2013, we were working in this district as part of a larger project investigating the preparations for NHI implementation. Although we had originally envisioned observing a set of management training activities, we instead followed over time an emergent bottom-up process to build systemic capacity for leadership development in this health district. Observing the diffusion of this bottom-up innovation allowed us to map key processes and steps that could be valuable in nurturing the emergence of systemic capacity for leadership development.

Our objective was to explore the emergent processes set in motion by an intervention that aimed at building systemic capacity for leadership, the mechanisms that facilitate them and the context in which such processes are effective.

We applied the realist evaluation (RE) approach and drew from theories relevant to understanding bottom-up processes of innovation and policy implementation. These address the activities of health workers at the frontline or periphery of a health system, who, in typically iterative processes, both re-interpret policies originating from the centre (or top) of the system and develop their own initiatives (Elmore, 1979; Lipsky, 1980; Hill and Hupe, 2002; Gilson *et al*., 2014; Gilson *et al*., 2018; Orgill and Gilson, 2018). In public sectors, innovation can be defined as ‘the introduction of new elements into a public service – in the form of new knowledge, a new organisation, and/or new management or processual skills, it represents discontinuity with the past’ (Osborne and Brown, 2005). We articulate bottom-up innovation as an initiative that is new and/or experienced as a new idea, practice or object and that is designed by actors at the bottom of the system (Brown and Osborne, 2012).

Our research question was ‘what are the mechanisms triggered by the Leadership Commission, and how does it interact with the existing social processes and norms, in the period 2013–15’?

## Methodology

We adopted RE, which seeks not only to answer whether an intervention (here the Leadership Commission Innovation) works (or not) but also to understand how and why it does so in a particular context. RE is driven by an understanding that interventions work (or not) because of the ways in which actors make sense of the intervention and react to it in specific contexts. We adopted RE because it is well adapted to study complex issues, such as how systemic leadership capacity results from emergent bottom-up processes. For many years, health policy and systems researchers have been making calls for methods to deal with complexity in health systems. While quasi-experimental studies are the foundation for assessing effectiveness and are very useful, unfortunately ‘the results of quasi-experimental studies do not identify in which conditions and through which configuration of factors the outcome is achieved’ (Marchal *et al*., 2012, p. 193). RE seeks to provide further insight for policymakers, asking the question: what about the intervention works and for whom, in what conditions and why? (Marchal *et al*., 2012).

Actor sensemaking^2^ is influenced by social or psychological drivers that are embedded in individual reasoning and/or are influenced by the context in which actors are embedded. These drivers are commonly referred to as generative mechanisms that trigger behaviours in actors in specific contexts, which shape the outcomes of interventions (Pawson and Tilley, 1997; Pawson, 2013). Using the intervention–context–actor–mechanism–outcome configuration (Marchal *et al*., 2019), we followed the RE cycle (Pawson and Tilley, 1997; Marchal *et al*., 2012). The steps in the cycle include the development of an initial programme theory (called PT1), which is then tested (the focus of this article) and refined. The latter refined PT (refined PT) can be used as the basis for future evaluation and lesson learning over time.

The PT1 (summarized in Box 2) was elicited through an initial round of interviews at the provincial and district level undertaken within the larger project in which this study was nested. This was called a context mapping phase and was conducted between late 2012 and early 2014, which included two site visits and 11 in-depth interviews with relevant provincial stakeholders and managers in the district. Programme Theory 1 (Box 2) was presented to and confirmed by the DM, this process allowed respondent validation of the analysis by the DM (Gilson, 2012). The conceptual underpinnings of PT1 are drawn from innovation theory, which recognizes that the assimilation of innovations into a system is often messy. Actors go through cycles of sensemaking along the journey to innovation adoption (Seligman, 2006), and the theory combines elements of diffusion and dissemination. Diffusion occurs when the ‘spread of innovations is unplanned, informal, decentralized, and largely horizontal or mediated by peers’ (Greenhalgh *et al*., 2004), while dissemination is a formal process, often centralized and occurring through vertical hierarchies, and perhaps including a communication strategy. This diffusion can occur through champions, vertical networks and boundary spanners. ‘Communication is a central part of sensemaking and organizing … a situation is talked into existence and the basis is laid for action to deal with it’ (Greenhalgh *et al*., 2004).

Box 2. **The initial programme theory (PT1)**

In a historically under-performing health district (C), that is an NHI pilot site (C) and where a shortage of leadership capacity has been identified (C), senior managers in the district will use their positional power and agency to design and implement bottom-up ideas and innovations to develop leadership capacity, because they perceive (M) the Minister’s call to strengthen management in districts as a priority, because they are motivated (M) and because they can generate resources to put plans into action (M). If the resulting bottom-up idea diffuses naturally and/or is disseminated through planned strategies that make use of champions, vertical networks, boundary spanners, sensemaking and sensegiving, a process of buy-in from relevant actors and a course of change will be triggered, which will lead to putting in place functioning structures and activities (proximal output) to support LCD in the local context. These activities will provide capacity development opportunities for managers, which will improve their competencies and contribute to a change in the behaviour of managers, ultimately improving the capacity within the district to manage and lead the district platform.

As the Leadership Commission Innovation had recently been initiated, we were able to gather information about the processes surrounding its implementation and so test our PT1. We did not observe long-term outcomes in terms of changed managerial behaviours in the 2 years in which we worked in the district. The process from ideation to proximal outputs (roles, structures and processes) itself took roughly one and a half years.

## Study design

We adopted a single case study design (Pawson and Tilley, 1997; Yin, 2014) to engage with the innovation as it unfolded and emerged in the real world (Gilson, 2012; Yin, 2014). As Yin (2014) notes, in case studies, theoretical propositions are desirable and serve as blueprints for the study: ‘such propositions will enable the complete research design to provide surprisingly strong guidance in determining the data to collection and the strategies for analysing the data’ (Yin, 2014). As such, it is compatible with RE and it is indeed often used as a study design in RE.

We defined our case as ‘the emergence of bottom-up innovation for leadership capacity development’ within a district context. We purposively selected the district of focus due to the presence of such an innovation and because the DM and district management team were willing to grant us access to the district. The district was also actively engaged in piloting reforms in preparation for a possible National Health Insurance system in the future. This was of relevance to the broader study and critically related to this RE. Districts (10 in total, out of 52 in South Africa) that were selected as National Health Insurance pilot sites were typically under-performing and identified as in need of system strengthening (Orgill *et al*., 2019). While some instructions on how to strengthen management were given as part of the large-scale policy reforms (e.g. hospital CEOs had to re-apply for their jobs as part of establishing fit for purpose), the DM also used her own long-standing contextual understanding of the district to inform their bottom-up approach to strengthening management capacity, thus going beyond programmatic interventions.

The organograms in the 52 health districts in South Africa are not all the same; however, they all include a DM with a district management team (Van Rensburg, 2012). In the district site, we worked with a district executive team made up of a DM and five senior managers. District management teams range from 12 to 20 people and include senior support service staff such as human resources, finance and transport, as well as senior staff from the sub-districts who oversee community health centres and clinics, regional hospitals, district hospitals, co-ordinators of local clinics and various senior programme managers and information officers (Dovey, 2002). The district in which we conducted research has four sub-districts.

## Data sources and data collection to test the programme theory

In late 2014–2015, we employed snowball sampling to identify actors who were involved in the design and implementation of the Leadership Commission in the district—following the trail of activities. In accordance with the focus of the IPT, we conducted 14 interviews with senior managers and mid-managers. We conducted semi-structured interviews focusing on how and why the intervention is working in relation to their specific place in the system, seeking to elicit assumptions underlying their actions and to understand the motivating factors that drive their behaviour, and we sought to understand their interpretation of outputs and outcomes and for whom it benefitted. We also kept field notes from our visits to the district, in which we recorded our observations, critical events and summaries of encounters with health workers. In 2015, in a 1-day workshop, we presented preliminary results to senior DMs to validate our observations.

## Data analysis

We employed thematic analysis in data analysis (Miles *et al*., 2014), using a staged process. We first created an MS Excel matrix for first-level coding, deductively applying the intervention–context–actor–mechanism–outcome configuration (Pawson and Tilley, 1997; Marchal *et al*., 2019) and drawing upon the core elements of the initial PT. Under ‘intervention’, we coded the aspects of the innovation, including key activities and processes, understanding of the problem (linked to assumptions) and understanding of the solutions (linked to outputs and outcomes). We stayed open to induction (Yin, 2014) in all the categories: as this was an emergent innovation, we could not easily predict what the data would reveal in all the categories. While we were looking for certain mechanisms, we stayed open to new activities, actors and subsequent mechanisms that would be triggered in the context.

In a second stage of analysis, we looked for patterns that matched the key elements of the programme theory (PT1). Specifically, we looked for generative mechanisms as defined earlier. We moved between the data of the interviews and notes and theory to derive the plausible intervention–context–actor–mechanism–outcome configurations, which are presented as findings in this paper (Miles *et al*., 2014). Two researchers who were both engaged in fieldwork in the district regularly checked interpretations with each other during the coding phase where we conjectured intervention–context–actor–mechanism–outcome configurations and during interpretation. We also discussed our findings with two additional researchers who worked on the larger project and who have published widely in the field of management and leadership in health systems in order to review the final conclusions. For further triangulation, in 2015 we conducted member checking of the findings at a 1-day feedback meeting with senior managers in the district to ensure the dependability of the data (Gilson, 2012). Refining the PT1 on the basis of empirical data is a way to enhance the analytical generalizability of case studies, lifting the data to a level of abstraction that allows conclusions to be transferable to other contexts (Robson, 2002; Gilson, 2012).

## Results

In this section, we present the main findings from our analysis. We start by describing the context and the innovation and then present the mechanisms triggered by the ‘Leadership Commission’ as it interacted with existing social processes and norms in the period late 2014–2015 to develop systemic capacity for LCD within the district. Please also see Supplementary Figure S1 for a visual presentation of key results.

### Initial context for LCD

In 2013, the district was a National Health Insurance pilot site, grappling with implementing a range of innovations as called for by the Minister of Health, including strengthening management capacity. The district had underperformed in terms of health outcomes relative to other districts in South Africa and faced challenges that included an under-resourced and under-staffed PHC platform. It was noted that some facilities in rural areas were only running with 50% of the staff they needed to fulfil their mandate,

*There are so many things that they have to do which were not in place when the structure* [of the district] *was developed, and the structure is actually not catching up with the new directives* (Participant 1, 2013).

Although only appointed in 2013, the new DM had 20 years of experience working in the district, with valuable institutional memory. She recognized the ways in which people grow within the health system and the need to support such growth for leadership development. As a participant noted,

*Some staff … they started off as pharmacy assistants; they died as deputy directors because of the growth through the systems and actually managing talent to make sure that we develop them* (Participant 1, 2013).

She was working with a strong District Executive Committee whose members all had high respect for each other. The DM also reflected that she had some exceptional managers in the district who had proven themselves through consistent performance in the health facilities they led. However, there were challenges. A District Executive Committee member expressed concern about the capacity of district management team members, especially in leadership skills (Participant 2, 2014):

*There were critical gaps that we noticed – not at District Executive Committee level but in the 30% [of DMT members] that I talked to you about, in terms of decision making; so we’re looking at issues like that: decision making as well as role modelling* (Participant 2, 2014).

Challenges at the lower level included operational managers who were not trained in administration and leadership. Additionally,

*We have a tendency of taking someone who is doing very well in a programme because of their professional expertise and throw[ing] them in the deep end for management and leadership and given the challenges that we are having presently, you need to do handholding because you are dealing with people and people break easily* (Participant 1, 2013).

Within the public system, district staff could access courses offered by the National Department of Public Service Administration (DPSA), including those addressing various levels of management development, such as leadership. The DM commented that she had seen a change in some of the operational managers who had graduated from these courses (Participant 1, 2013).

However, as access to these programmes was via nomination through the Provincial Department of Health, it cannot be assumed that all managers accessed them. The Human Resource Department, meanwhile, asserted that their role was to develop management skills, such as how to discipline staff, rather than broader leadership skills. There was, then, limited bureaucratic infrastructure to support LCD within the system and there were no additional resources for bottom-up innovation. There was, however, a fairly new Regional Training Centre (RTC) in the district, although solely funded for human immunodeficiency virus (HIV)/acquired immune deficiency syndrome (AIDS) training in 2013.

### Bottom-up innovation for LCD

The Minister’s call for management development and the DM’s contextual understanding of the need for leadership skills in the district triggered a sensemaking process. This sensemaking was driven by the DM’s firm belief in leadership as an important practice and set of skills that managers need to fulfil their duties; she wanted managers to be prepared for future leadership roles. This belief was shared by the DEC:

*And we are very excited, you know that we never focused on this issue [leadership] [before], so we are really doing our best as the DEC to make sure that one day when we are no longer here, these are the managers that are reporting under us must be able to take the hospital and primary healthcare facilities to another level* (Participant 2, 2014).

The DEC identified leadership as one of six strategic priorities (with HIV, AIDS and tuberculosis, information, communications and technology, staff wellness, quality and national core standards) for the district at the beginning of 2013, establishing thematic commissions for each.

The DM, using her positional power and acting as champion, showed expressed commitment in her use of language and narratives to prioritize the issue (Fox *et al*., 2015) by establishing the ‘Leadership Commission’ as a strategic priority in a district—this was a new organizational arrangement. Although no specific definition of leadership was used, the narratives clearly spoke to issues of interdependence and leadership for the future. The DM also showed institutional commitment, beyond expressed commitment, by setting up the basic bureaucratic infrastructure to address the issue (Fox *et al*., 2011). She did this by drawing on resources in the district, including establishing the Leadership Task Team, to drive the work of the Leadership Commission:

*It’s … difficult to make people think out of the box. You see, when we started with the LTT people were saying ‘Let’s do the things we are supposed to do.’ and we said we have been trying that for quite some time and it’s not helping, let’s do something different. Let’s shift the focus from ourselves [from] what we need to do as leadership [which] is to build relationships, let us equip these people [managers] with skills so that when we leave it should be easier for someone to take over, there must not be a skills gap* (Participant 1, 2015).

### What mechanisms underlay the successful setting-up and functioning of the Leadership Task Team?

The DM reflected on a personality assessment which had identified her as someone with ‘vision’, who liked to take ‘action’ and who liked structure but had fallen short on ‘interdependence’. Consequently, she had intentionally brought her focus towards sharing responsibility and allowing others to drive change alongside her. Based on this self-reflection, the DM purposefully engaged two highly motivated District Executive Committee members who believed in the need for LCD in the district, to lead the Leadership Task Team. Specifically, selected because of their proven leadership capabilities, both had many years of experience working as managers in the district and both were CEOs of well-performing hospitals. A shared understanding of what leadership entails was evidenced in the Leadership Task Team leaders’ narratives about their own experience of leading and being led.

There were no additional resources for the Leadership Commission. Yet, the new priority status for LCD provided legitimacy for the Leadership Task Team’s establishment and for attracting human resources to it. This unleashed the personal capacity of the two leaders. Their high motivation, linked to their positive leadership experiences and their sensemaking in leadership, helped to establish the Leadership Task Team successfully. The sensemaking of Leader 1 of the Leadership Task Team was also influenced by a Department of Public Service and Administration leadership development training programme that she had completed:

*It is through the various courses that I did. You know what makes me to be more interested in leadership is the fact that in 2013, I think, 2013-2014, I was involved in the Executive Development Programme’… So now, my mini research was on leadership. I focused, you know there is a strategic challenge that you must give – so I took that as my strategic challenge to say that I do want to see one day the leadership being in place even if one day we leave the Department of Health, so that the hospital must keep on running* (Participant 2, 2014).

The two leaders selected six additional Leadership Task Team members from their work networks. They developed a clear allocation of roles and responsibilities in the team and identified champions for specific tasks. Leadership Task Team members expressed confidence in the LTT leadership. They held regular meetings every second month and there was shared sensemaking around the need for hard work to bring about change:

*It was a team effort. It was a team effort, madam, what we do is a team effort. We sit and say: ‘This is fine’. We present, we task each other. You go and do, you two you go and develop the tool, you go and do that, and next meeting you present, and we critique* (Participant 3, 2014).

*We are a very diligent group, you know. [The leader] is a [hard] driver* (laughs) (Participant 4, 2015).

Given that the Leadership Task Team leaders and some members were part of the District Executive Committee, the Leadership Task Team became integrated into the routine management structures in the district. Accountability structures were also put in place as each of the six commissions had to present on progress at the annual strategic planning forum or ‘lekgotla’. This accountability facilitated sensegiving on the priority status of all of the commissions.

While the Leadership Task Team was functioning well at the district level as a team with champions, one respondent noted that the structure was not yet reaching lower down in the system. Another manager reflected that working on LCD was time-consuming as leadership could be understood as something that was not a priority, given other pressures and job responsibilities:

*You running a lot of balls, you running a training plan for leadership, you running the HAST training plan, you running the [training form the] equitable share [budget] … You know, it’s a lot of things [but] you have some success* (Participant 4, 2015).

Within the Leadership Task Team, there were also different interpretations of what ‘leadership’ meant. One manager understood it as ‘emotional intelligence’—a narrow understanding that suggests sensemaking was not fully comprehensive among all actors.

### The Climate Survey as part of systemic capacity building for LCD

A key event in which the Leadership Task Team engaged between 2013 and 2015 was the development and application of a climate survey in the district. There was no routine method of gathering information about the experiences of managers in the district or their capacity needs. The survey was administered to managers of all levels of seniority within the district, as well as to specialist health professionals. On district management and leadership, they were asked (1) what they wanted to be changed, (2) what did not need changing, (3) how they felt about their senior managers and (4) the general challenges they experienced.

The mechanisms that enabled the development of the climate survey included collective sensemaking about the need for information to drive the Leadership Commission forward and the diffusion of ideas and resources between the DM and one Leadership Task Team leader, whose existing capacity was unlocked to support systemic capacity building for LCD. The leader had completed the Department of Public Service and Administration Executive Development Programme and had previously developed a climate survey to assess leadership capacity and skills gaps in her own hospital. The earlier survey tool was updated as the basis for the district-wide climate survey.

Furthermore, institutional commitment was shown by setting up the processes for conducting the climate survey and for sharing the results. It was conducted district-wide using halls or other convenient venues where managers could complete the survey. The process of administering the survey and the results of the survey triggered self-reflection by the senior managers. Engagement with the results served as a moment of sensemaking for the DEC, both about gaps in their own leadership and management as well as in the district at the sub-district levels:

*[The climate survey] was a massive eye opener, people were very unhappy with the status of management and leadership in the district* (Participant 1, 2015).

Key challenges identified included a lack of decision-making at the middle management level, a lack of role models, not meeting goals and targets and a lack of initiative. The District Executive Committee identified serious challenges with ∼30% of managers in the district:

*There were gaps in terms of professional appearance and issues of performance in terms of initiative, and meeting the goals and targets. Those things are very important for any leader. We talk about issues of professional ethics and work ethics because if [indistinct] you are a latecomer as a leader you influence your people … the other issues were issues like team building, it’s a whole lot of issues* (Participant 2, 2014).

Other challenges identified included few development opportunities, the lack of management visibility in the district (at all levels) and overall levels of staff wellness. Staff wellness was also identified as one of the six wider district priorities. The survey showed that managers sometimes did not feel rewarded, only called in when something negative happens and that ‘no one ever says what you do right’ (Participant 4, member of the LTT, 2015). However, one manager commented that the DM was actively trying to change that situation through her own behaviours in meetings.

The survey results were disaggregated by facility and presented to the District Executive Committee, then to the wider district management team and, finally, via vertical networks to sub-districts. The planned dissemination of the results was part of the process of sensemaking and sensegiving at all levels of the district:

*And we developed, we analysed that and we went back and presented to say there, our the people are saying regarding management et cetera, … and we said X Hospital, this is your climate survey, so out of this you develop a plan. The LAMs [local area managers] are developing the plans for the clinics and progress must be shared quarterly* (Participant 3, 2014).

After the presentation of the findings in the sub-districts, individuals were nominated to be responsible for the leadership mandate at lower levels of the system as part of the dissemination strategy. They acted as champions, developed bottom-up plans and sent a progress report to the Leadership Task Team leader. The Leadership Task Team leader commented that this ensured accountability by the people developing the plans.

### The RTC as part of system capacity for LCD and the role of boundary spanning

Another input into the development of the system capacity for LCD was made by the RTC and, more specifically, the manager of the RTC who was selected as a member of the Leadership Task Team. The RTC manager was highly motivated to support training generally in the district as this was her passion, and the DM had noted to her personally that she could come directly to her for support. She also had a firm grasp of training structures in both province and district. She was highly dedicated and well respected by the leaders of the Leadership Task Team and had ‘a wonderful team’ (Participant 3, 2014).

The district was an NHI pilot site with many external service providers working towards strengthening the PHC platform. While the RTC was only given a budget for HIV training, she was given a mandate by the DM to extend the RTC’s services towards workplace skills more broadly. In her boundary-spanning role across vertical and horizontal networks in the district, the manager used bargaining and negotiation to leverage^3^ external service providers to offer free training for managers for a fixed time as part of LCD. For example, she negotiated with one service provider, hoping to do research in the district, to extend their change management training to a group of supervisors:

*They can have research here, but then we want one workshop just for the supervisors also. So that we don’t have to pay private companies for training […], you know, emotional intelligence [training] is R143 000 (gasps) for 20 people and then it’s a two-day workshop and then I still [have to] pay the catering* (Participant 4, 2015).

An additional two service providers were persuaded to use the results of the climate survey to support managers with targeted training. A leader of the Leadership Task Team commented:

*Part of the intervention from our task team was that we are struggling with a, b, c, d. [… and we identified] those managers that are not doing well. So, yes, we had targeted training in terms of leadership gaps* (Participant 2, 2014).

Training was also provided internally. For example, the larger hospital management teams developed plans for computer skills training to address a particular need of some of their operational managers.

There was, thus, a combination of external training and internal training for LCD. However, building structural capacity requires thinking about longevity and sustainability. Challenges that arose included that the service providers were all externally funded and would eventually leave the district. The RTC manager noted that, ideally, she should have a set of trainers who could take over these roles, but she had neither the people nor the funding for such activities. A ‘training the trainer’ process was undertaken to try and circumvent this problem, using staff in the district to replace the external service providers. However, these staff members still had other jobs and it was sometimes difficult to ask their managers to release staff to conduct training:

*Person X tried to train people before she went away but those people have other work. … As long as you train a trainer who still has another job, one of the jobs is gonna suffer, either your job or the one you have been trained at, and of course it is the training that suffers* (Participant 4, 2015).

## Discussion

This paper reports a bottom-up innovation that focused on building systemic capacity for leadership development within a DHS, providing insights about how leadership development can be held and nurtured as an internally driven system capacity development intervention. We sought to draw out and synthesize findings on the mechanisms triggered by bottom-up innovations for LCD and how they interact with existing social processes and norms.

Potter’s capacity pyramid provides a useful lens to describe the proximal outputs that were achieved. He describes a four-tier interdependent hierarchy of areas relevant when building capacity in systems, including ‘(A) structures, systems and roles, (B) staff and facilities, (C) skills, and (D) tools’ (Potter and Brough, 2004). The establishment of a functioning Leadership Task Team (output) as a key structure for decision-making for LCD was part of the emergent process of enacting the ‘Leadership Commission’ as a strategic priority in the district. This structure harnessed the collective capabilities of a key team of people, drawing on individual competencies that already existed in the district context. The climate survey was central in enabling the use of information for decision-making (output) in a context where there was previously no information on the leadership needs of managers. The survey was used to understand the capacity needs of managers and for planning leadership development activities. Using disaggregated survey results, it also informed the identification and tasking of sub-district champions to develop bottom-up plans for LCD. The harnessing of information at the right time for the right purpose is part of the capacity (Potter and Brough, 2004) needed for systemic capacity building. While the regional training centre was initially only developed for HIV training, through the RTC manager and DM, it became a key part of the support service capacity for LCD (output) in the district. This included the provision of free management and leadership training by three external service providers as a starting point for LCD, and an annual training plan (covering many areas beyond HIV) for the district, developed by the RTC manager in consultation with the Provincial government. These experiences resonate with Baser and Morgan’s definition of organizational capacity, as comprising individual contributions and the collective capabilities of teams and people working together (Baser and Morgan, 2008).

We conclude that PT1 did materialize: senior managers used their positional authority and agency to support LCD for managers through boundary spanners, champions and vertical networks, as we explain further.

When we refined PT1, we identified the importance of prioritizing management capacity development as part of health system strengthening reforms by the Minister of Health (Pillay and Barron, 2012) as a key feature of context that triggered sensemaking by the DM on ways to develop management capacity, In this case, the DM specifically focused on setting up systemic capacity for leadership development. We also identified that buy-in from actors is not sufficient to build systemic capacity for leadership development, one needs a group of people who have shared beliefs and are highly motivated to drive leadership development, as leadership development is not typically prioritized in the health system. Gilson and Daire (2011) noted that there is growing interest in South Africa in developing the management competencies of health managers but they noted key challenges for the institutionalization of LCD in South Africa: (1) there is a limited national conversation on ‘the nature of leadership required to re-engineer primary health care (PHC) and implement NHI, or coordinated and coherent strategies of leadership development’ (p. 2), (2) while management and leadership are related, they are not the same and leadership needs to be thought about explicitly in the context of managers who can vision in and navigate complex health systems and (3) human resource departments need to develop more strategic and sustained approaches to developing leadership across the system, including for those without titles. There is also a current focus on formal training, but leadership development requires workplace-based learning and non-formal learning approaches (peer-based learning) that allow for the practice of leadership development (Gilson and Daire, 2011).

### The Leadership Commission within its context

Given these realities, the Leadership Commission did not fit neatly into the usual health system processes and there was limited bureaucratic infrastructure for LCD. The first step in building systemic capacity for LCD was identifying leadership as a strategic priority at the highest level of the district. Importantly, this was a show of expressed commitment (Fox *et al*., 2011) by the DM, triggered by her sensemaking, her champion role, and her newly acquired positional power in the bureaucracy (Greenhalgh *et al*., 2004; Sriram *et al.*, 2018). This step was supported by shared sensemaking with her five senior managers about the need for leadership development in the district to sustain the system and was driven by a deep historical and localized understanding of the context, including the need for leadership development of managers in the district management team and in lower level managers.

Unlike policies implemented in a top-down manner, bottom-up ideas diffuse in a system when the ‘spread of innovations is unplanned, informal, decentralized, and largely horizontal or mediated by peers’ (Greenhalgh *et al*., 2004). Once ideas become more formalized, intentional dissemination of information begins. As there was no formal mechanism for gathering information on leadership development needs or how to develop leadership capacity initially, the use of tacit knowledge was a key mechanism shaping the bottom-up innovation. Such knowledge was complemented by the climate survey, an information-gathering exercise implemented through vertical networks, which stimulated personal reflection by senior managers about how staff viewed them. It was used both to disseminate knowledge about the Leadership Commission and to set up planning processes for leadership development at the sub-district level. The champions for leadership development drew on their past experiences of leading and relevant training (Orgill *et al*., 2019). Identifying the right individuals and harnessing their competencies was a key mechanism that enabled systemic capacity development for LCD—for bottom-up innovation, it is important to harness readily available resources given wider constraints. Key mechanisms included shared beliefs and a shared motivation by these senior managers to develop future leaders and address current leadership behavioural gaps. Dalakoura (2010) identified similar features associated with leadership development in organizations, such as (1) the need for a steady focus on developing leaders at all levels, (2) an organizational culture that values leadership behaviour at all levels and (3) leadership development being seen as a priority of strategic importance.

The DM’s show of institutional commitment enabled systemic capacity building for LCD. ‘[T]he process of setting up the basic “institutions” and bureaucratic infrastructure to mount a response or act beyond expressed commitment only’ (Fox *et al*., 2011) helps to ‘lock in’ a response to prevent new programmes being undone or uncoupled from the system. Beyond building individual role capacity (e.g. the LTT leaders), when implementing innovations, it is also important to focus on developing structural and system capacity (Potter and Brough, 2004). Administrative and organizational arrangements need to be in place so that there are proper fora for decision-making and functioning systems to ensure that programmes/services can be provided for the long term (Cassels and Janovsky, 1991; Potter and Brough, 2004; Orgill *et al*., 2021). The DM’s ability to take a systems perspective was a key mechanism that enabled thinking across the DHS towards developing systemic capacity for LCD. Moreover, as the LTT leaders were among the senior managers within the DMT, a routine management infrastructure, the priority status of leadership development was maintained through the collective sensemaking in this team with high power in the district. Information gathering through vertical networks up and down the system using a climate survey served as a useful tool to gather information and to disseminate knowledge down the system about the LTT and the Leadership Commission. As Orgill *et al*. (Orgill *et al*., 2021) have argued: ‘building the capacity of the “structures” (e.g. meetings, organisational processes) that hold the district health system [is] critical for developing capacity and unleashing the tools, skills and infrastructure in the system at large’. Unlike policies implemented in a top-down manner, bottom-up ideas diffuse in a system when the ‘spread of innovations is unplanned, informal, decentralized, and largely horizontal or mediated by peers’ (Greenhalgh et al., 2004). Once ideas become more formalized, intentional dissemination of information starts to occur.

### Insights derived from this experience for future LCD at the district level in South Africa

Our results confirm findings from other studies. Health system challenges are becoming increasingly complex and require intersectoral and interdependent solutions. Management without leadership simply will not be enough (Kwamie, 2015). In terms of lessons learned for policy and practice, our study suggests that institutionalizing systemic capacity for LCD from the bottom-only is challenging.

Central-level governmental support will be needed for such efforts by prioritizing LCD through additional resource allocation and by developing bureaucratic infrastructure to support district-led LCD. This infrastructure could include developing the capacity of public training centres to provide ongoing leadership training and support. The National Academy for health leadership and management in South Africa could also be resurrected, drawing from the experience of the NHS Leadership Academy in the UK (NHS England online, 2023). The South African Academy was launched in 2012 but has never since been formalized as a structure within government (Bateman, 2013). If this Academy offered direct support to bureaucratic structures in districts that seek to strengthen governance and leadership as part of their PHC agenda and mandate, this would itself reflect an expressed and institutional commitment to systems leadership development. Finally, extending human resource departments’ training mandate to include leadership development as part of a workplace-based learning strategy might also foster change (Schaay *et al*., 1998; Gilson and Daire, 2011). Overall, central government should identify and harness the capacity of managers and teams who embody systems’ leadership characteristics to drive the development of these foundations in the health system (Kwamie, 2015; Gilson *et al*., 2023).

The systems leadership literature ‘is quite diverse, but there is a common focus on the critical processes and practices needed to nurture change within complex, interconnected and interdependent systems’ (Gilson *et al*., 2023). In this district, they effectively adopted a workplace-based learning approach. Adopting a workplace-based learning approach to LCD may enable an embedded approach to LCD that addresses the interconnected nature of leadership and can support the development of people and teams, whose new leadership practices work to change the system from within (Blanchard and Carpenter, 2012; Choonara *et al*., 2017; Cleary *et al*., 2018). Furthermore, developing leadership capacity will require maintaining a balance between individual and organizational capacity strengthening, recognizing the two as complementary (Dovey, 2002; Doherty *et al*., 2018). Indeed, leadership practice can support the creation of enabling environments for wider systems change (Kwamie, 2015). One way to develop this complementary balance is to expose individuals to systemic learning opportunities—such as drawing on systems leadership principles and practices in coursework and linking this to system-wide processes, such as intersectoral action; and embracing pedagogies that include workplace-based learning (Gilson *et al*., 2023), which will itself require support from high-level health systems leadership. Johnson *et al*. (Johnson *et al*., 2021) provide lessons for the sustainability of all health LDPs: (1) ensure self-sufficiency with domestic funding; (2) deliver through national or regional institutions; (3) draw on national or regional faculty; (4) ensure country ownership; (5) train participants to become future mentors and (6) anticipate resource constraints in the setting (Johnson *et al*., 2021).

## Conclusions

We found that building systemic capacity for LCD requires prioritization of ‘leadership’ by those with positional authority and that bottom-up processes of LCD require strong institutional commitment.

While there is increasing recognition that district-level leadership is a critical component of a well-functioning health system, many health systems do not yet have the systemic capacity to either hold and sustain LCD initiatives or think beyond individual training programmes. Recognizing leadership as a district priority and establishing the associated bureaucratic infrastructure and budget commitment are essential first steps. Internal leadership can, moreover, be harnessed to drive wider LCD, especially among those who have experienced positive leadership and believe in the need for leadership development. While new resources will be needed, creative opportunities and leverage points should be integrated and synthesized to support LCD. Opportunities and leverage points could include embedding LCD initiatives (e.g. improved decision-making and information sharing) within local health system strengthening efforts, for example when strengthening clinical governance structures, to institutionalize new behaviours and capacities within these structures. Alternatively, leveraging large-scale health system strengthening initiatives by embedding LCD within them could secure funding support for, e.g., individual and team competency development through action-learning approaches.

## Supplementary Material

czae099_Supp

## Data Availability

The data reported in this paper are not publicly available as such consent was not obtained.

## Footnotes

1. A National Health Insurance had not at this time been formally adopted or implemented in South Africa.

2. Sensemaking has to do with the way managers understand, interpret and create sense for themselves based on the information surrounding … strategic change (Rouleau, 2005, p. 1415).

3. The definition of leverage used here is ‘to use something that you already have in order to achieve something new or better’ https://dictionary.cambridge.org/dictionary/english/leverage.

## Reflexivity statement

Three of the four authors were living and working as health policy and systems researchers in South Africa at the time of data collection. Although they have a good understanding of the national context and debates around DHS development and health leadership, the research was conducted in areas outside their usual base, and none have prior experience as managers in the South African public health system. This may have introduced some bias into their understanding of the specific local settings and experiences. The fourth author is based outside South Africa and hence brought international experience and insights into the analysis that may have also introduced some biases in interpretation. The authorial team played different but inter-linked roles as outlined in the Author contribution section.
