# Supplementary material for: Initiating systemic capacity development for leadership from the bottom-up: a realist evaluation of a leadership innovation in a South African health district
Source: Health Policy Plan. 2024 Oct 30;40(1):31–41. doi: 10.1093/heapol/czae099 (PMC11724640; doi:10.1093/heapol/czae099)
Supplement: czae099_Supp [file czae099_supp.zip › suppl_data/Supplementary figure 1 Key results through the lens of the Intervention- Context-Actor-Mechanism-Outcome configuration.docx]

**Context**

- Management capacity development prioritised by National Ministry of Health
- Leadership development not typically prioritised in the health system
- Need for leadership development amongst different levels of managers identified in the district
- Limited infrastructure for leadership capacity development in the district
- Newly appointed district manager has many years’ of experience in the district and has good relationships with her district executive team

Supplementary figure 1: Key results through the lens of the Intervention- Context-Actor-Mechanism-Outcome configuration

**Actors:**

- District Manager
- District Executive team (top five managers)
- District Management Team
- Sub-district champions

**Mechanisms (resources and reasoning)**

- Tacit knowledge and system thinking capacity of the DM who had a strong belief in leadership development
- Deployment of the positional authority of the DM to establish new structures
- Excitement and motivation in the team to focus on leadership development
- Sensegiving and shared sensemaking
- Developed and delegated leadership of a Leadership Task Team to highly motivated and high performing members of the district executive committee
- Tacit and formal training knowledge of the leaders of the LTT
- Expressed commitment of leadership as a priority in the district
- Institutional commitment by leveraging existing structures and resources in the district and setting up new structures

**Changed situation (proximal outcomes)**

Systemic capacity for leadership development:

- Foundations in place to support leadership capacity development in the district:
- Established leadership roles of the LTT
- Functioning LTT
- Developed and implemented a climate survey to understand leadership needs
- Harnessing of information to develop strategies and plans for LCD
- The regional training centre leveraged as resource for LCD

**Initial situation**

No formal bureaucratic structures and processes that specifically targeted and/or budgeted for Leadership Capacity development in the district.

The bottom-up ***innovation*** considered in this case is the *Leadership Commission*. This Commission was not a structure, but rather an expressed statement signalling the importance of leadership capacity development.

The original premise of the Leadership Commission was to develop the capacity of individuals to lead, but over time it was recognised that the systemic capacity at the local level to implement LCD initiatives was limited. There were no formal structures and processes in place that specifically targeted and/or budgeted for LCD. Subsequently, efforts were made to set up structures and processes in the district to give ‘life’ to the Leadership Commission.
